# Correlates of COVID-19 Pandemic on Anxiety among Adults in Appalachia, USA

**DOI:** 10.34172/jrhs.2021.68

**Published:** 2021-11-14

**Authors:** Ram Lakhan, Louisa Summers, David Tataw, Peter Hackbert, Manoj Sharma

**Affiliations:** ^1^Department of Health and Human Performance, Berea College, Berea, KY, USA; ^2^Department of Allied Health, Northern Kentucky University, Highland Heights, KY, USA; ^3^Department of General Studies, Berea College, Berea, KY, USA; ^4^Department of Environmental and Occupational Health, University of Nevada, Las Vegas, NV, USA

**Keywords:** Anxiety, COVID-19, Pandemic

## Abstract

**Background:** Anxiety problems have increased in the COVID-19 pandemic worldwide. However, very little is known about the anxiety rates in the new normal phase of the disease when adults have been assumed to be adjusted. The study aimed to find out the difference in anxiety in a convenience sample of Appalachian adults during the new normal phase of the COVID-19 pandemic, examine its association with sociodemographic factors, and compare it with the anxiety levels before the pandemic as recalled by the participants.

**Study design:** A cross-sectional study.

**Methods:** The Generalized Anxiety Disorder-7 scale was used in the present study. The Chi-square test was used to examine the difference between the severity of anxiety before and during the new normal phase of the COVID-19 pandemic in terms of sociodemographic and behavioral correlates. Pearson correlation was used to see the strength of the association between anxiety and age.

**Results:** Although the anxiety rate was stabilized by the time people approached the new normal phase of the COVID-19 pandemic, its severity increased significantly among those with preexisting anxiety (*P*=0.001). Anxiety was found highly associated with female and minority gender, student status, lower education and income level, marital status, cohabitation with parents, and cigarette consumption (*P*=0.001). A slight inverse association was observed between age and anxiety before and during the new normal phase of the COVID-19 pandemic (*P*=0.001).

**Conclusion:** The young and females seem to be suffering from a higher burden of anxiety. Research is suggested to identify ways to develop social support-based community programs to address this issue.

## Introduction

According to the American Psychological Association, anxiety is an emotion characterized by feelings of tension, worried thoughts, and physical changes, such as increased blood pressure^1^. Anxiety among people over 18 years of age, females, and males is reported to be 19.1%, 23.4%, and 14.3%, respectively. Moreover, about 31.1% of people experience some form of anxiety disorder in their lives, out of whom 22.8% and 33.7% suffer from serious and moderate impairment in the USA^1,2^. Unaddressed anxiety can continue to become severe and chronic and in turn, affect the quality of life^3^. Severe and chronic anxiety can lead to depression and trigger more severe health issues, such as cardiovascular diseases^4^ and diabetes^5^.

The COVID-19 pandemic created big chaos in the social-ecological system and affected the functioning of every person at micro, meso, exo, macro, and chrono levels ^6,7,8^. Individuals, families, and organizations modified their living and working policies and created a new system to keep everyone safe. This change, along with other factors, such as the fear and experience of infection with the virus, the possibility of death, potential loss of income, uncertainty about vaccine and treatment, and working in settings with a potentially higher chance of exposure to virus acted as strong triggers for anxiety in the populations^9^.

The escalation in the rate of anxiety and other mental health issues, including stress, depression, and sleep problems were recognized, and worldwide cross-sectional studies were carried out to estimate the burden^10^. The prevalence of anxiety during the earlier phase of the COVID-19 pandemic was reported as high as 37.4%, 28%, and 11.5%-17% in China^11^, India^12^, and Italy^13^, respectively. A systematic review and meta-analysis of 14 studies published in July 2020 reported the anxiety prevalence of 32.9% (95% CI: 28.2, 37.9), 23.8% (95% CI: 16.2, 33.5), and 33.7% (95% CI: 27.5, 40.6) in Asia, Europe, and the overall general population, respectively^14^. A recently published systematic review reported anxiety rates between 6.33% and 50.9% in the general population^15^. The catastrophic changes in social situations were due to the increased risk of chronic anxiety disorders caused by COVID-19 ^16,17^.

It is also known that people overcome anxiety-related symptoms from a crisis and triggering situation as time passes, and they adjust with the situation^18^. Often substances, such as cigarettes, tobacco, and alcohol are used for coping^19^. Previously conducted studies have provided estimates of anxiety problems in populations at the beginning of the pandemic^20,21^. As time passed, psychological resilience and coping flexibility helped people adjust to the new environment of the ongoing pandemic^22^. Around Fall 2020, people started returning to their occupations with precautions, calling this phase a “new normal”^23^. By August 2020, over 23.5 million people were infected with the virus. The death toll was over 800,000 people^24^.

 This study aimed to estimate the occurrence of anxiety in a convenience sample of a college population, including students, staff, and faculty members in the Appalachia region in the USA, during the new normal phase of the COVID-19 pandemic, examine its association with sociodemographic factors, and compare it with the anxiety levels before the pandemic, as recalled by the participants.

## Methods

This study followed a cross-sectional study research design. The research proposal was approved by the Berea College Institutional Review Board, KY, USA (Protocol #452). Berea College students, its staff, and faculty members constituted the survey population. Berea College is a tuition-free institution that primarily serves the lower socioeconomic population of the USA and other countries. Students come mainly from the Appalachia region, which is regarded as an economically poorer area of the USA^25^. All participants were provided a written informed consent form to sign electronically before proceeding with the survey. The sampling was conducted using a convenience sampling approach. The inclusion criteria included students and other adults aged 18 years and older who were associated with the college and lived in the Appalachia region surrounding Berea College at the time of the survey. Students and any other members associated with college under the age of 18 were excluded from the study. Berea College has a community of over 2,000 people (including all students, staff, and faculty members). A survey with a Qualtrics link was sent to all of them electronically. The Qualtrics link was also posted on Facebook for the Berea College community to take the survey. The data were collected from August 25, 2020, to September 30, 2020. The survey was sent on August 24, 2020, with a follow-up request in the second week of September 2020.

The Generalized Anxiety Disorder (GAD-7) scale was used for the identification and classification of anxiety severity. The GAD-7 is a widely used standardized, reliable, and valid scale. It has a sensitivity of 89% and a specificity of 82%^26^. The scale has 7 self-reported Likert-type items and assesses defining symptoms of GAD present in the past two weeks. These items are rated on a 4-point scale in which the items “not at all” =0, “several days”=1, “more than half the days”=2, and “nearly every day”=3. These items assess the feeling of nervousness, anxiousness (or being on edge), worrying too much about different things, and ability to relax. The seven items included 1) feeling nervous, anxious, or on edge, 2) not able to stop or control worrying, 3) worrying too much about different things, 4) trouble relaxing, 5) being so restless that it is hard to sit still, 6) becoming easily annoyed or irritable, 7) feeling afraid as if something awful might happen. The total score of the scale ranged from 0 to 21, and higher scores indicated more severe GAD. The scale has been proven to be effective even in identifying three other common anxiety disorders, including panic disorder, social anxiety disorder, and post-traumatic stress^26,27^. The cut-off score of 5, 10, and 15 points demonstrated mild, moderate, and severe anxiety, respectively.

The survey questionnaire also included items on sociodemographic and behavioral factors, including smoking and drinking. Participants were asked to rate GAD-7 items twice considering their present and past status as recalled. The difference in GAD-7 scores between current and past recalled status provided the statistics of people with anxiety. The questionnaire was sent to 10 people before and after uploading on Qualtrics for checking the link and flow of questions. A sample size of 384 was found suitable for a descriptive study, considering normal approximation to the binomial calculation, significance level (type I error) =0.05, a total width of confidence interval (CI)=0.1, and CI=95% ^28^.

 The Chi-square test was used to see the difference in the prevalence and severity of anxiety before and during the COVID-19 pandemic. Prevalence of anxiety during the new normal phase was presented in two categories by combining minimal and mild and moderate to severe degrees of anxiety. The Chi-square test was used to see the difference between milder and severe categories of anxiety in terms of sociodemographic and behavioral factors. Age, gender, country of origin, ethnicity, student status, education level, household income, marital status, cohabitation with a partner, having children, employment status, place of living, and living arrangement were considered sociodemographic factors, while chronic condition, infection with COVID-19, and smoking and drinking status were regarded as behavioral factors. Only five categories of ethnicity (Caucasian/White, African American, Latino or Hispanic, Asian, and Others) were considered in this study. Student status was categorized as full-time and part-time/not active students. Students who decided to take a semester off were included in the part-time/ not active category. Moreover, the education level of participants was presented with four education categories (associate or below, undergraduate, master’s, and doctorate/professional). Eventually, six categories of household income (lost income due to COVID-19, less than $25,000; within the range of $25,000- $50,000; $50,000- $100,000; $100,000 and more; and prefer not to say) were considered in this study. Association with age was examined using the Pearson correlation. P-value less than or equal to 0.05 (P≤0.05) was considered statistically significant.

## Results

A total of 572 people responded to the survey, out of which 349 (61%), 132 (23.1%), and 12 (2.1%) individuals identified themselves as female, male, and other genders. The mean ±SD age of participants was 29.49+15.03 years. Out of the total 572 participants included in this study, 277 (48.4%), 95 (16.6%), and 200 (35.0%) individuals were in the age range of 18-25 years, 26-50 years, and 51 years and older, respectively. About 303 (53%) and 183 (32.5%) individuals considered themselves full-time and part-time students, respectively. The United States was the country of origin for 427 (74.7%) participants, and 55 (9.6%) participants were international students from 37 countries around the world. Among all participants, 343 (60%) and 48 (8.6%) persons considered themselves Caucasian or White and African American, respectively. Moreover, 334 (58.4%) participants had an undergraduate level of education or less, and 162 (28.3%) participants earned less than $25,000 annual income. The unmarried, married, divorced or separated participants constituted 56.8% (n=325), 24.5% (n=140), and 4.0% (n=23) of the study population. In addition, 158 (27.6%) and 322 (56.3%) participants lived with a partner and alone, respectively. The individuals with children and without children constituted 20.8% (n=119) and 64.9% (n=371) of participants. In total, 203 (35.5%) and 279 (48.9%) participants lived in urban and rural settings, respectively. Furthermore, 220 (38.5%), 129 (22.9%), and 131(22.9%) individuals lived with their parents, rented the house, and owned the place of living, respectively. Moreover, 64 (11.2%) participants had some type of chronic disease, and 64 (11.2%) participants had someone infected with COVID-19 in their families or their close circle. Eventually, 38 (6.6%) and 187 (32.7%) participants identified themselves as smokers and alcohol users, respectively (Table 1).

**Table 1:** Difference in anxiety with sociodemographic and behavioral factors during the new normal phase of COVID-19 pandemic

**Sociodemographic factor**	**Total**		**Prevalence anxiety**				**X**^2^	***P*** **-value**
			**Minimal to Mild**		**Moderate to Severe**			
	**Number**	**Percent**	**Number**	**Percent**	**Number**	**Percent**		
Age (yr)							28.54	0.001
18 -25 (young)	277	48.4	101	40.6	148	59.6		
26-50 (middle-aged)	95	16.6	50	56.8	38	43.2		
≥51 (older)	200	35.0	78	70.3	33	29.7		
Gender							17.11	0.001
Female	349	61.0	145	46.6	166	53.4		
Male	132	23.1	82	65.1	44	34.9		
Others	11	2.1	2	18.2	9	81.8		
Country of origin							0.101	0.751
International	55	9.6	22	48.9	23	51.1		
USA	427	74.7	203	51.4	192	8.6		
Ethnicity							2.455	0.653
Caucasian/ white	343	60.0	162	51.4	153	48.6		
African American	49	8.6	19	45.2	23	54.8		
Latino or Hispanic	47	8.2	19	45.2	23	54.8		
Asian	13	2.3	7	58.3	5	41.7		
Other	39	6.8	22	59.5	15	40.5		
Student status							45.51	0.001
Full time	303	53.0	104	38.4	167	61.6		
Part time / not active student	183	32.5	125	71.0	51	29.0		
Education							19.57	0.001
Associate and below	41	7.2	24	66.7	12	33.3		
Undergraduate	334	58.4	132	43.7	170	56.3		
Master	66	11.5	42	66.7	21	33.3		
Doctorate and professional	44	7.7	28	65.1	15	34.9		
Household income							36.97	0.001
Lost due to COVID-19	12	2.1	5	50.0	5	50.0		
Less than $25,000	162	28.3	49	34.0	95	66.0		
$25,000 - $50,000	106	18.5	49	49.5	50	50.5		
$50,000 - $100,000	103	18.0	59	59.6	40	40.4		
$100,000 and more	42	7.3	32	82.1	7	17.9		
Prefer not to say	58	10.1	32	61.5	20	38.5		
Marital status							32.34	0.001
Unmarried	325	56.8	122	41.9	169	58.1		
Married	140	24.5	95	71.4	38	28.6		
Divorced /Separated	23	4.0	9	42.9	12	57.1		
Living with a partner							20.56	0.001
No	322	56.3	124	42.9	165	57.1		
Yes	158	27.6	98	65.8	51	34.2		
Having children							34.68	0.001
No	371	64.9	143	42.9	190	57.1		
Yes	119	20.8	86	74.8	29	25.2		
Employment status							0.38	0.267
Employed (full or part)	236	41.3	143	64.4	79	35.6		
Unemployed	17	3.0	8	53.3	7	46.7		
Place of living							0.46	0.258
Rural	279	48.8	127	49.4	130	50.6		
Urban	203	35.5	98	53.0	87	47.0		
Living arrangement							35.50	0.001
Living with parents/family	220	38.5	81	40.5	119	59.5		
Owned	131	22.9	92	73.0	34	27.0		
Rented	129	22.6	52	44.1	66	55.9		
Chronic condition							0.33	0.181
No	327	57.2	171	52.5	155	47.5		
Yes	64	11.2	56	47.1	63	52.9		
COVID-19 infection							0.21	0.373
No	384	67.1	197	51.6	185	48.4		
Yes	64	11.2	31	48.4	33	51.6		
Smoking status							4.69	0.021
Non-smokers	409	71.5	214	52.6	193	47.4		
Smokers	38	6.6	13	34.2	25	65.8		
Alcohol consumption							0.71	0.224
Non-drinkers	258	45.1	135	52.7	121	47.3		
Drinkers	187	32.7	91	48.7	96	51.3		

The severity of anxiety was affected by such variables as gender, age groups (young, middle-aged, and older), student status (full time vs part-time), education and income level, marital status, living with a partner, having children, living arrangement, and smoking status (*P =*0.001). Ethnicity, employment status, place of living (rural vs urban), contract with COVID-19, chronic conditions, and drinking status did not affect the severity of anxiety (*P*=0.063) (Table 1).

The severity of anxiety in participants was found to be different before and during the COVID-19 pandemic. In addition, preexisting minimal to mild anxiety was associated with severe anxiety during the COVID-19 pandemic (χ^2^=79.83, P=0.001) (Table 2). Anxiety was mildly associated with age before (R^2^= - 0.273, P=0.001) and during new normal phase of COVID-19 (R^2^=- 0.316, *P*=0.001).

**Table 2:** Difference in anxiety prevalence and severity before COVID-19 and during the new normal phase of COVID-19 pandemic (X^2^=79.83, P-value=0.001)

**Level of anxiety**	**Pre-COVID-19**		**During COVID-19**	
	**Number**	**Percent**	**Number**	**Percent**
Minimal	183	32.0	100	17.5
Mild	147	25.7	129	22.6
Moderate	85	14.9	97	17.0
Severe	34	5.9	122	21.3
Total	449	78.8	448	78.3

## Discussion

This study aimed to estimate the occurrence of anxiety in a convenience sample of Appalachian adults during the new normal phase of the COVID-19 pandemic, examine its association with sociodemographic factors, and compare it with anxiety levels before the pandemic as recalled by the participants. The findings of this study offer a new understanding of anxiety and its correlates in the Appalachian region during the new normal phase (August-September 2020) of the COVID-19 pandemic. Several studies have indicated a steep increase in anxiety problems worldwide^29^, while this study showed a higher rate of anxiety in the Appalachian adults before the pandemic which did not differ much during the later stage of the pandemic. However, the anxiety problems intensified in people with preexisting anxiety, which lends credence to the hypothesis that people with preexisting anxiety are more susceptible to chronic and severe anxiety in the COVID-19 pandemic. Analyzing the psychological impact of COVID-19 among the elderly population in China and made corresponding suggestions^30,31^. People who identified themselves as other than male and female were found to be experiencing a higher burden of anxiety followed by the female gender. Studies around the world also found the same pattern of higher rates of anxiety among women^31,32,33^ and people who do not identify themselves as either men or women (non-binary)^35,36,37^. Large-scale population studies have also identified the female gender as the most potent predictor of post-traumatic stress disorder after a pandemic^38^. The COVID-19 pandemic was also found to be a strong factor for intensifying mental health issues in the population^33^.

There was no difference between people of U.S. origin and other nationalities in terms of the anxiety rates. Pandemic has created chaos in people’s lives worldwide; however, the U.S. has a better system in place for addressing such situations. Accordingly, the international participants who were worried about their families in their respective countries at that time might have felt more secure in the U.S. for themselves. This feeling of security might explain the lack of a greater sense of anxiety in these people, compared to their American counterparts. At the beginning of the pandemic, public health experts expressed serious concerns about mental health issues among international students^39^. Academic institutions recognized the vulnerability of international students to mental health issues and tried to provide the needed support to these students, which might explain the lower rate of anxiety among international participants, compared to their American counterparts.

Irrespective of the mode of learning (in person, online, or hybrid), full-time students were found to suffer from a higher rate of anxiety than those who were continuing their education part-time. The results of a study conducted by Hoyt et al. (2020)^36^ on 707 U.S. students indicated a higher rate of perceived stress and general anxiety. A lower rate of anxiety in part-time students can be attributed to the availability of social support and socialization opportunities. Moreover, part-time students often live with their families and friends and hold some level of employment within the community. These living and working settings offer more freedom and interaction opportunities for social interaction. In France, researchers observed an increase in severity of anxiety and stress among students who lived in almost confined situations and had fewer opportunities for interaction, compared to those who moved out from the universities and lived with their parents^40^.

Participants with an undergraduate level of education experienced higher anxiety. Students who were pursuing an undergraduate degree were also included in this category and their student status was associated with the greater burden of anxiety. In general, people with less than undergraduate education have a lower level of income and financial challenges, which were exaggerated in the pandemic and became major triggers^34^.

People with an annual income of $25,000 followed by those with an income range of $25,000-$50,000 experienced a higher burden of anxiety, compared to those with higher income. These findings were consistent with international studies^41^. The pandemic affected the regular income of people, especially those working in low-earning jobs. Mainly, low earning people work for essential services; however, this population does not earn enough to support themselves with a regular income, and going to work has been associated with the risk and fear of contracting the virus. Low income and lessened earning opportunities due to unemployment might have contributed to anxiety in these people, while people unemployed from better-paying jobs might have received a good amount of unemployment support from the government that might have kept them relatively less affected with anxiety.

Similar to full-time students, people who were unmarried, divorced, separated, or living single experienced a greater burden of anxiety than those who were married or living with a partner. These findings suggest that being married or living with a partner help reduce the risk for anxiety. Similarly, having kids was also found to be an anxiety-reducing factor. Several studies assessed the effect of social support on anxiety during COVID-19 and found a low level of support associated with a higher prevalence of anxiety disorder (OR=3.18, 95% CI: 2.54-3.98)^42^. Özmete and Pak (2020)^43^ have also found mediating effect of social support on anxiety during the COVID-19 pandemic^43,44^.

Living arrangement was found to be associated with the higher occurrence of anxiety for those who lived with their parents and for the people who lived in a rented house, compared to those who owned their place of living. Living with parents indicates toward a younger population. It was known that COVID-19 mortality risk is less associated with younger age; therefore, probably the younger people living with their parents were more frequently allowed to engage in outdoor essential activities including employment^45^. Low-income families of college students experienced food insecurity that had put pressure on college students to work outside^46^.

The anxiety scores did not differ between people with and without chronic conditions. It could be due to fact that right from the beginning information was spread that people with chronic conditions were at higher risk of suffering from COVID-19. A mechanism to protect older and diseased people was followed at all levels in society. These people might have followed better protective measures and as time passed during the pandemic, they might have felt less threatened by COVID-19. People who smoked were found to have a greater burden of anxiety, compared to non-smokers. Research has already proven a higher rate of anxiety among smokers^33^, which may explain the greater burden of anxiety in this population during the COVID-19 pandemic.

Based on the obtained results, age was inversely associated with anxiety, and people in the age group of 18-25 years were suffering from the greatest burden of anxiety during the COVID-19 pandemic. These findings were in line with those obtained in other studies^34^. According to the census 2019, 23.3%, 28.2%, and 9.1% of students in the USA complete their high school, bachelor’s, and master’s education by the age of 25 years, respectively^47^. After graduation, people get into employment and start building their families. Presumably, people older than 25 may have income, family, and kids which might have played a protective role against occurrence as well as the escalation of anxiety^44^. Few studies conducted in Europe did not find a difference in anxiety between the young and older population^41^, while other studies reported higher anxiety in older people^48^.

Findings suggest that further studies are needed to identify ways to increase social networks for younger people, especially the student population. Probably, interventions based on a social-ecological framework should be given importance in designing and implementing community-level interventions for reducing the severity and risk of anxiety during the challenging times of the COVID-19 pandemic^6,7,49^.

***Strengths and Limitations:*** This study provides some understanding of the correlates associated with higher anxiety scores in Appalachian adults. Additionally, it can provide a comparative view of anxiety before and during the new normal phase of the COVID-19 pandemic. However, regarding the limitations of the present study one can refer to self-reporting, recall bias, cross-sectional study design, and small sample size.

## Conclusion

 The findings of this study are very helpful in understanding the risk of increased anxiety among Appalachian adults with preexisting anxiety. In addition, the findings also suggest that adults in Appalachia are adjusting to the new normal, and that anxiety is not increasing at a similar rate as it was found at the beginning of the pandemic around the world. The anxiety was also associated with the female and minority populations and to a lesser extent with younger age.

## Acknowledgments

 Authors sincerely thank their institutions for carrying out this research, participants for their participation, and Margaret Skylar Gayhart for the post-survey link on social media and spreading words among students for taking this survey.

## Funding

 None.

 Highlights

- Anxiety rate was stabilized by the new normal phase of the COVID-19 pandemic
- Severity of anxiety increased among those with preexisting anxiety
- Anxiety was found highly associated with female and minority genders and full-time students
